# Dexamethasone as endocrine disruptor; type I and type II (anti) oestrogenic actions on the ovary and uterus of adult Wistar rats (*Rattus Novergicus*)

**DOI:** 10.5935/1518-0557.20180061

**Published:** 2018

**Authors:** Joseph Babatunde Dare, Babajide Arogundade, Olakunle Oladipupo Awoniyi, Adebiyi Aderinola Adegoke, Damilare Adedayo Adekomi

**Affiliations:** 1 Anatomy Department, Faculty of Basic Medical Sciences, College of Health Sciences, Osun State University, Osogbo, Nigeria

**Keywords:** dexamethasone, ovary, uterus, anti-oestrogenic, endocrine disruptors and stress

## Abstract

**Objective:**

Dexamethasone is a widely used glucocorticoid, which has been prescribed increasingly in recent years. The effects of Dexamethasone on the ovary and uterus was investigated in present study.

**Methods:**

Twenty (20) adult female Wistar rats, weighing 130-170 g were assigned to four (4) groups of five (5) animals each. The rats in the control group received saline, while the rats in the experimental group was subjected to oral treatment of dexamethasone of 12 mg/kg, 10 mg/kg, and 7 mg/kg doses daily for a period of 10 days, respectively. The rats were slaughtered after 24 hours of the last administration, and the uterus and ovaries were harvested following abdominal incision. Histological and biochemical investigations were carried out and the results were analyzed using ANOVA with the Graph-Pad prism software package 6.

**Results:**

There was a significant decrease in the activities of the carbohydrate metabolic enzymes of the uterus in the dexamethasone-treated groups compared to the control group (*p*<0.05). Vacuolation, atrophy, thick epithelium, enlarged cells, inactive interstitial glands and follicular cyst, characterized the histological observation in the dexamethasone-treated groups in a dose-dependent manner.

**Conclusion:**

This present study revealed that high-dose dexamethasone causes multiple changes in the histological features of the ovary and uterus, exerting type I and type II anti-oestrogenic effects on the female reproductive compartment.

## INTRODUCTION

The female reproductive system consists of three major components, the ovary, uterine tube and uterus, which function synchronously under strict hormonal control, in a graded continuum, from fully competent menstrual cycles (eumenorrhoea) to the complete absence of cyclic ovarian activity (amenorrhea) (Agarwal *et al*., 2005; Tsigos & Chrousos, 1994). Psychological stressors affect the reproductive axis, causing it to depart from eumenorrhoea and move towards amenorrhea (Bloom *et al*., 2001; Inoue *et al*., 2003; Klein *et al*., 2001). Studies have confirmed the existence of reproductive suppression mechanisms on the hormonal pathways, through which several isolated, intense stressors impact the reproductive function (Sugama & Conti, 2008; Tarín, 1996; Brady *et al*., 2016). However, the mechanisms through which stress suppresses reproduction in humans are the major focus of current research (Cymerman & Rock, 1994).

Glucocorticoids (GCs) classified as corticosteroids are an example of steroid hormones. Glucocorticoids bind to a receptor called glucocorticoid receptor (GR) (Pelt, 2011). These receptors are present in vertebrate animal cells. Glucocorticoids (glucose + cortex + steroid) regulate glucose metabolism, and it is synthetized in the adrenal cortex (Chrousos *et al*., 1993). It is also known that glucocorticosteroids form part of the feedback mechanism in the immune system, suppressing the immune function, consequently leading to inflammation. GCs are used in medicine to treat diseases caused by an overactive immune system, such as allergies, asthma, autoimmune diseases, and sepsis (Liu *et al*., 2010; 2011). GCs have many diverse (pleiotropic) effects, including potentially harmful side effects (Matsuwaki, 2004). GCs have been reported to interfere with abnormal cell division that take place in the cancer cell formation mechanism; therefore high doses of GCs are used in the treatment of cancer (Pelt, 2011). This includes inhibitory effects on lymphocyte proliferation, as in the treatment of lymphomas and leukemia; and the mitigation of side effects of anticancer drugs (Provan *et al*., 2010)

GCs bind to the glucocorticoid receptor (GR), and activate the GR complex, which in turn, up-regulates the expression of anti-inflammatory proteins in the nucleus, and reduces the expression of pro inflammatory proteins in the cytosol by preventing the translocation of other transcription factors from the cytosol into the nucleus (trans-repression) (Koch *et al*., 2000, Leung & Bloom, 2003). Glucocorticoid specific receptors, target cells, and effects distinguished GCs from mineralocorticoids and sex steroids. However, corticosteroid includes both glucocorticoids and mineralocorticoids (produced by the adrenal cortex). Glucocorticoids are secreted in the zona fasciculata of the adrenal cortex, whereas mineralocorticoids are synthesized in the zona glomerulosa (Sugino *et al*., 2004). Cortisol, also known as hydrocortisone, is the most important human glucocorticoid ever known. It is essential for survival, and it regulates or supports cardiovascular, metabolic, immunologic, and homeostatic functions. It is worth of note that various synthetic glucocorticoids are available; these are used either as replacement therapy in glucocorticoid deficiency or to suppress the immune system (Lupien *et al.,* 2009).

Dexamethasone is part of the synthetic glucocorticoid that suppresses the immune system, being 20-30 times stronger than hydrocortisone. It binds more tightly to the glucocorticoid receptor than cortisol does (Gao *et al.,* 2003; Carlson, 2010). Glucocorticoid therapy is often limited by several adverse reactions associated with GC excess. Excess GC results in growth retardation in children; immunosuppression; cardiovascular disorders like hypertension and atherosclerosis; osteoporosis; myopathy; and diabetes mellitus. Currently, attention is focused on oxidative stress as one of the major determinants of endothelial dysfunction and cardiovascular senescence. The main reason for all unwanted effects of GC is that dexamethasone induces the overproduction of reactive oxygen species, causing unbalance of physiological processes (Matsuwaki *et al*., 2004).

Studies have shown that the response to stress is usually accompanied by the hyperactivation of the hypothalamic-pituitary-adrenal (HPA) axis. Female hypothalamus contains higher concentration of corticotrophin-releasing hormone (CRH) than their male counterpart; therefore, the HPA axis reaction to stress is higher in females than in males. Stress is a potent activator of CRH release from the hypothalamus and extra-hypothalamic sites (Pazirandeh *et al.,* 2002). CRH type-I receptor knockout mice have shown a deficient ability to mount an effective stress response. A direct neural connection exists between CRH and GnRH; CRH is the major regulator of the HPA axis and the CRH-induced proopiomelanocortin peptide, such as β-endorphin, reduces the hypothalamic GnRH pulse generator and concurrently inhibits GnRH secretion. The decrease in pulsatile release of LH subsequently leads to anovulation and interruption of endometrial decasualization and pregnancy wastage (Mazerbourg *et al.,* 2003). Dexamethasone, as a corticosteroid, is used in the treatment of rheumatic problems, a number of skin diseases, severe allergies, asthma, chronic obstructive lung disease and brain swelling (Leung *et al.,* 1997). Therefore, the study aimed at investigating the impact of dexamethasone on the ovary and uterus of adult Wistar rats.

## MATERIALS AND METHODS

### Animal Care and Management

Twenty (20) adult Female Wistar rats, weighing 130-170 g were procured from the College of Health Sciences Animal House, Osun State University, Osogbo. The ethical approval on animal act right was obtained from the Institutional Animal Care Committee of the same Institution. The animals were randomly divided into four (4) groups with each group comprising of five (n=5) rats. They were kept in the Laboratory for two (2) weeks for acclimatization and were fed on a standard diet (Vital Feeds and Grand Cereals Ltd); water was given *ad libitum* and maintained under standard conditions. The animal room was well ventilated with a temperature range of 25-27ºC under day/night in a 12-12 h photoperiodicity. All the experimental procedures were done following the experimental guidelines of the Institutional Animal Ethics Committee (IAEC) of the Osun State University, Osogbo campus, Osun State.

### Administration

Twenty (20) rats with an average weight of 130g were used in this experiment and were subdivided into 4 groups 1, 2, 3, and 4, of five (5) animals. The animals in group 1 received only distilled water and were tagged the control group. Those in groups 2, 3, and 4 received different doses of dexamethasone. Freshly prepared dosages of dexamethasone were administered each day of the experiment, by dissolving 30 mg of dexamethasone in 50 ml of distilled water. The animals in group 2 received 12 mg/kg/body weight of dexamethasone (DEX). Groups 3 and group 4 received 10 mg/kg and 7mg/kg/body weight respectively of dexamethasone (DEX). The drug was administered orally, using a metal oral cannula. The treatment lasted for seven (7) days and was carried out at 07:00 hours daily.

### Animal slaughtering

The animals were euthanized 24 hours after the last administration. Their uteruses and ovaries were excised following an abdominal incision, and were later fixed in Bouin's fluid for histological analysis using H/E and PAS stains. The uterus was also homogenized in 5% sucrose solution in cold ice for determining the Glucose-6-phosphate dehydrogenase (G-6-PDH) activity in Tissue Homogenate using the method of Charmandari *et al*. (2008).

### Histological Techniques

Histological examination was carried out on the tissues fixed in Bouin's fluid. Tissue blocks were sectioned for routine Hematoxylin and Eosin (H&E) The fixed organs were cut in slabs of about 0.5cm thick transversely and transferred to 70% alcohol for dehydration. The tissues were passed through 90% and absolute alcohol and xylene for different durations before they were transferred into two changes of molten paraffin wax for 1 hour each in an oven at 65ºC for infiltration. They were subsequently embedded and serial sections using a rotary microtome at six microns (6µ). The tissues were transferred onto albumenized slides and allowed to dry on a hot plate for 2 minutes. The slides were dewaxed with xylene and passed through absolute alcohol (2 changes); 70% alcohol, 50% alcohol and then to water for 5 minutes. The slides were then stained with hematoxylin and eosin.

### Statistical Analysis

The results were expressed as Mean ± standard error of mean (SEM), and subjected to statistical analysis using the ANOVA Graph-Pad prism software package 6 for data analysis.

## RESULTS

**Figure 1:**
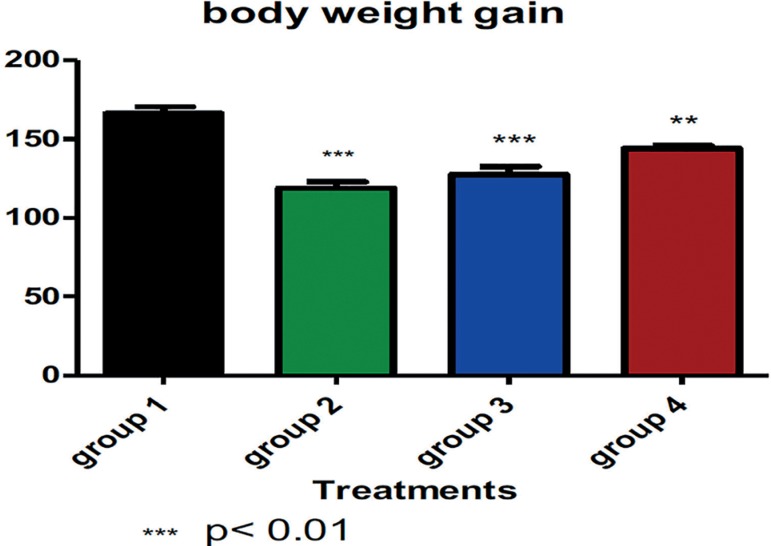
The average animal body weight gain in each group. G-6-PD enzyme activity was significantly reduced in the animals that were treated with dexamethasone. It was observed that rats treated with 12 mg/kg/body weight of dexamethasone demonstrated higher significant reduced G-6-PD enzyme activities relative to the control and rats treated with 10 mg/kg and 7 mg/kg/body weight of dexamethasone as shown in Figure 2.

**Figure 2:**
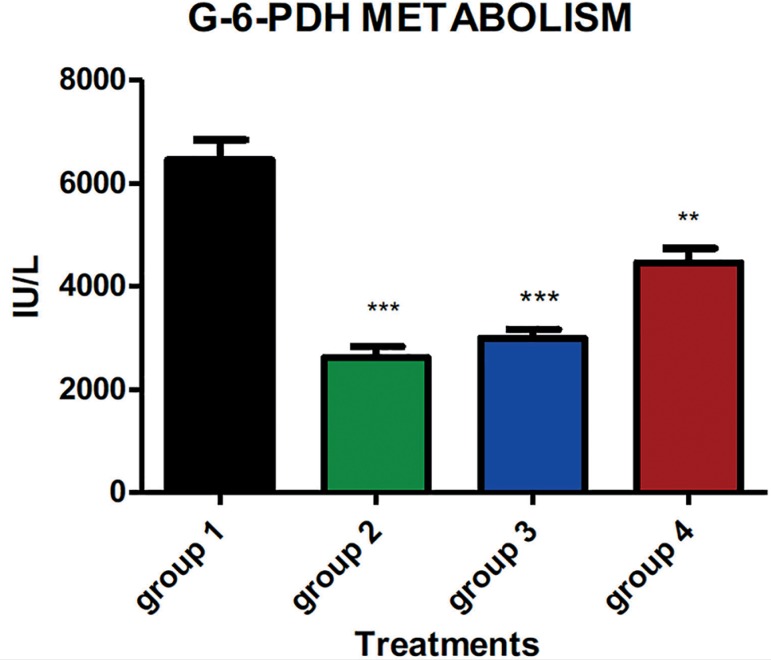
The result obtained shows a decrease in the activities of the action of carbohydrate metabolic enzymes of the uterus in the groups treated with dexamethasone.

Significant reduction in animal bodyweight was noticed across the groups. However, the animals treated with 12 mg/kg body weight demonstrated higher reduction in their weight when compared to their control counterparts' *p*<0.01. Reduction in bodyweight was dose-dependent, as shown in the Figure 1 below. Figure 2 demonstrates G-6-PDH enzyme activity following treatment with different doses of dexamethasone-exogenous substances, at low doses, G-6-PDH enzymes' activities were reduced as the concentration of the enzymes were lower in the uteruses of the treated animals. Therefore, dexamethasone exerts reduction effects on carbohydrate metabolism enzymes even at lower doses. This reduction in G-6-PDH enzyme activity was more pronounced and significant at lower doses than at higher doses.

**Plates 1 A and B:**
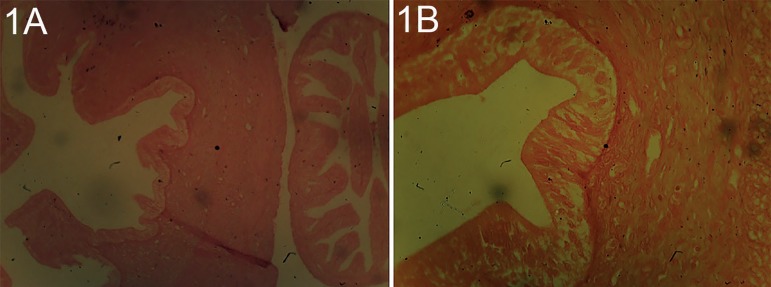
Uterine session of a rat from the control group stain with H/E X 100 and X400. The three (3) layers of the endometrium, myometrium and perimetrium were distinct. Characterized with tall columnar cells at the epithelia lining, showing the basic features of the uterus; the two distinct layers of endometrium and myometrium. The endometrium classically revealed the brush border and the tall columnar epithelium lining the inner layer of endometrium. The endometrium two functional layers, the luminal stratum and the basal stratum basalis were shown.

**Plates 2 A and B:**
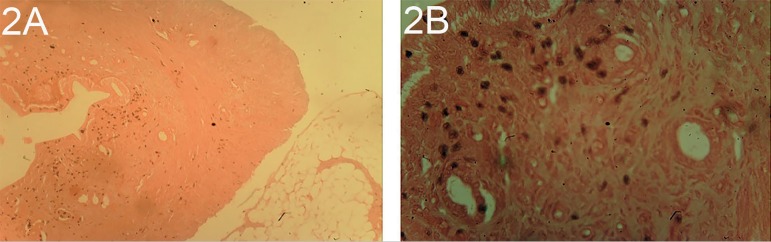
Uterine session of rat treated with 7 mg/kg/body weight of dexamethasone stain with H/E X100 and X400 shows thickened luminal and glandular epithelium with increased number of enlarged cells. Thin, atrophic endometrial and glandular epithelium consisting of low columnar cells. Sparse endometrial glands and atrophic myometrium.

The histological observation of the uterus of the control animals demonstrated tall columnar cells lining the endometrium. The endometrium classically revealed the brush border and the tall columnar epithelium lining, the inner layer of the endometrium. The endometrium's two functional layers, the luminal stratum and the basal stratum basalis were demonstrated on Plate 1. However, the uterine sessions of rats treated with 7 mg/kg/body weight of dexamethasone showed thickened luminal and/glandular epithelium with increased number of enlarged cells. Thin, atrophic endometrial and glandular epithelium consisting of low columnar cells, with sparse endometrial glands and atrophic myometrium, which are characteristic manifestation of type I anti-oestrogenic effects of endocrine disruptors' (Plate 2).

**Plates 3A and B:**
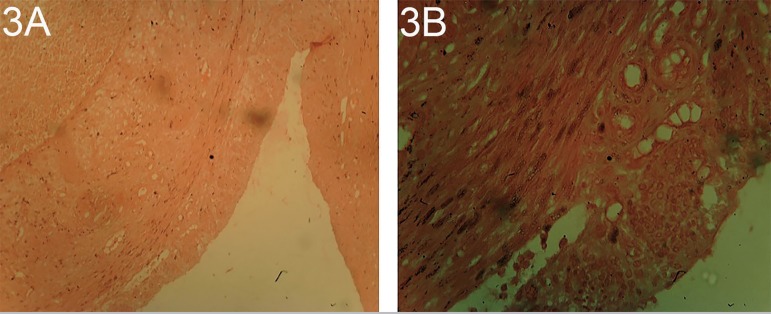
Uterine section of rats treated with 10 mg/kg/body weight of dexamethasone stain with H/E X100 and X400 shows thickened luminal and glandular epithelium with increased number of enlarged cells, attenuated, low columnar luminal and glandular epithelium and sparse endometrial glands, atrophic myometrium, hyperplasia, hypertrophy of luminal and glandular epithelium with cystic endometrial glands.

**Plates 4A and B:**
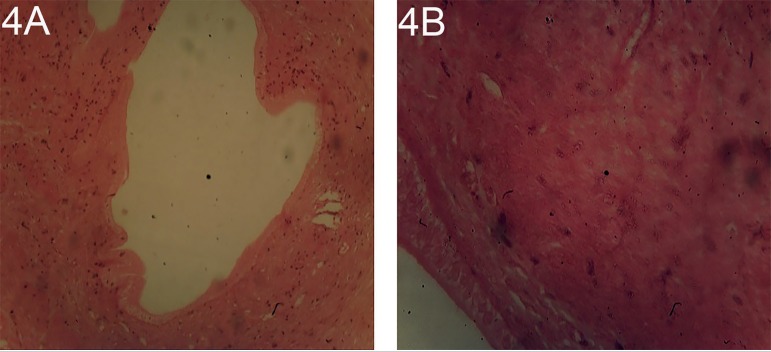
Uterine section of rats treated with 12 mg/kg/body weight of dexamethasone stain with H/E X100 and X400 Shows dilation/cystic change in endometrial glands, Neutrophil infiltration of endometrium, transformation of luminal/glandular columnar epithelium into a stratified squamous epithelium and squamous metaplasia present in luminal and glandular epithelia.

The uterine sections of the rats treated with 10 and 12 mg/kg/body weight of dexamethasone revealed thickened luminal and/glandular epithelium with increased number of enlarged cells, attenuated, low columnar luminal and glandular epithelium and sparse endometrial glands and atrophic myometrium, hyperplasia and hypertrophy of luminal and glandular epithelium, and cystic endometrial glands. In the animals treated with 12 mg/kg/body weight of dexamethasone dilation/cystic change in endometrial glands, neutrophil infiltration of endometrium, transformation of luminal/glandular columnar epithelium into a stratified squamous epithelium and squamous metaplasia present in luminal and glandular epithelium were well-expressed, these are observable features that classified type II anti-oestrogenic effects on the uteruses' (Plates 3 and 4).

**Plates 5A and B:**
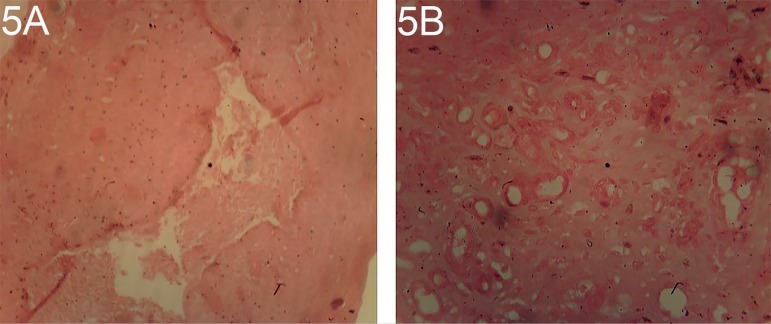
Ovarian section of a rat treated with 10 mg/kg/body weight of dexamethasone, stained with H/E X100 and X400. Atrophy inactive interstitial glands, reduced numbers of Follicles and corpora luteal, follicular cysts and stromal cell hypertrophy/hyperplasia.

**Plates 6 A and B:**
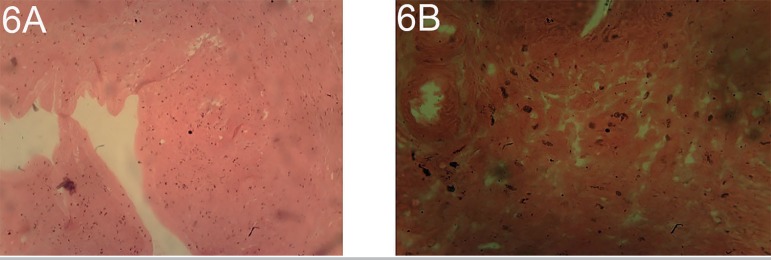
Ovarian session of a rat treated with 12 mg/kg/body weight of dexamethasone stained with H/E X100 and X400, with atrophic inactive interstitial gland and interstitial stromal cell hypertrophy/hyperplasia, reduced numbers of Follicles and corpora luteal and follicular cysts.

Ovarian histopathological observation showed at 10 and 12 mg/kg/body weight of dexamethasone atrophy inactive interstitial gland and interstitial stromal cell hypertrophy/hyperplasia, reduced numbers of Follicles and corpora luteal and follicular cysts accompanied by pathological changes or histological alterations observed in the uterine compartment as depicted on Plates 5 and 6.

## DISCUSSION

This study has demonstrated a decrease in bodyweight and G-6-PDH enzyme activities by the administration of dexamethasone in a dose-dependent manner, with the highest significant reduction observed at 12 mg/kg (*p*<0.05). The studies by Rhen & Cidlowski (2005) and Revollo & Cidlowski (2009) indicated that dexamethasone changes the glucocorticoid stimulant and reactive gene expression in the nuclei of ovarian cells under the regulation of glucocorticoid. Song *et al*. (2011) studied the effects of dexamethasone on Bax protein expression as an apoptotic protein in the germ cells of 35 female mice, and found that glucocorticoid compounds such as dexamethasone can come up with apoptosis and disruption in oogenesis processes affecting pro-apoptosis proteins such as Bax. And, in this study, dexamethasone has led to damage to uterine tissue. In the present study, the administration of Dexamethasone resulted in atrophy and inactive interstitial glands, reduced numbers of Follicles and corpora luteal, follicular cysts and the histological profile of the uteruses showed dilation/cystic changes in endometrial glands, transformation of luminal/glandular columnar epithelium into a stratified squamous epithelium.

As expressed in type I anti-oestrogenic morphological responses associated with endocrine disruption, thin, atrophic endometrial and glandular epithelium consisting of low columnar cells and sparse endometrial glands and atrophic myometrium (Yuan, 1991; Goldman *et al*., 2000) as well as in the ovary, endocrine disruption cause atrophy with inactive interstitial glands; glandular cells are small and spindle-shaped and associated cystic anovulatory follicle present.

**Plates 7 A and B:**
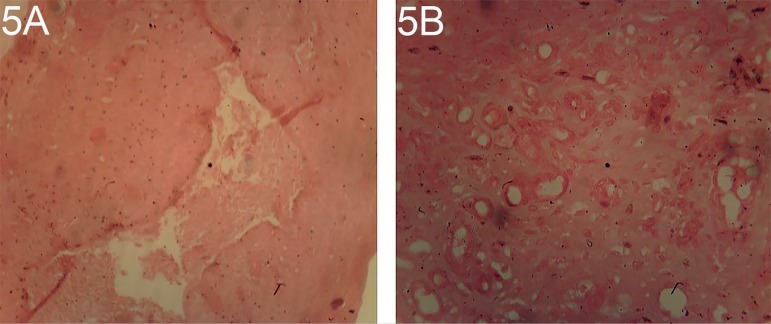
Ovarian section of rats treated with 7 mg/kg/body weight of dexamethasone stained with H/E X100 and X400. Degenerating follicles, with oocyte cell death, Cyst (C) formation within a corpus Luteum (CL) and vacuolation of the ovarian cortex. It also shows an obvious reduction in the number of follicles and corpora luteal, this characterizes ovarian atrophy.

This study has expressed parallel observations among the animals that were administered with dexamethasone even in a dose-dependent manner. Therefore, in line with the medications of the histological actions of endocrine disruptors, as described by Yuan (1991), dexamethasone could be classified as an endocrine disruptor, producing type I anti-oestrogenic effects on the ovary and uterus of animal models. Probable reasons could be attributed to aromatase inhibition, which impairs the conversion of androgens (produced by theca interna cells) to estrogen by the follicular zona granulosa. Endogenous production of ovarian estrogen is thus reduced, initiating widespread atrophic reproductive tract changes. Modulation of feedback control on the hypothalamus and pituitary by reduced endogenous estrogen levels suppress gonadotrophin secretion, resulting in the formation of follicular cysts from anovulatory follicles (Yuan, 1991; Li & Davies, 2007). This type I anti-oestrogenic effect was clearly expressed in the ovaries and uteruses of the animals treated with 7 mg/kg/body weight of dexamethasone as shown on Plates 2 and 7.

Dexamethasone also caused degeneration in follicles, with oocyte death and ovarian cortex vacuolation. Reduction in the number of follicles and corpora luteal, which characterizes ovarian atrophy, were observed in the animals treated with 10 mg/kg and 12 mg/kg/body weight of dexamethasone. Hyperplasia and hypertrophy of luminal and glandular epithelium of the uterus was noticed in animals treated with higher doses of dexamethasone.

These observations characterized the type II anti-oestrogenic effect, as revealed by Yuan (1991) and Li & Davies (2007). The administration of dexamethasone may result in negative feedback at the hypothalamic-pituitary-gonadal axis, inhibiting gonadotrophin release, consequently inducing ovarian atrophy and hyperplasia in the lining columnar and glandular epithelia, as per shown on Plates 4 and 6. Pazirandeh *et al.* (2002) stated that corticotrophin releasing hormones (CRH), the major regulator of the HPA axis, induced the proopiomelanocortin peptide, such as β-endorphin, which reduce the hypothalamic GnRH pulse generator activity and concurrently inhibits GnRH secretion. This results in decrease pulsatile release of LH, subsequently leading to anovulation, interruption of endometrial decidualization and pregnancy wastage (Pazirandeh *et al.,* 2002; Kim *et al*., 2002).

## CONCLUSION

The results of the present study indicates that the administration of high doses of dexamethasone resulted in changes to the histological features of the ovary and uteruses of adult female rats, typical of type I and type II anti-oestrogenic effects. The administration of dexamethasone resulted in decreased activities of carbohydrate enzymes of the uterus metabolism, and reduction in body weight.

### Recommendation

This study has established a positive correlation in the stressor action, using a dexamethasone model and the female infertility among the adult Wistar rats. It is therefore, pertinent to extrapolate from relevant studies that will make use of human subjects, within the limits of the ethics as a model study to evaluate antifertility, promoting effects of the endocrine disruptors of which dexamethasone is part.
